# Complete genome sequence of lytic bacteriophage RG-2014 that infects the multidrug resistant bacterium *Delftia tsuruhatensis* ARB-1

**DOI:** 10.1186/s40793-017-0290-y

**Published:** 2017-12-18

**Authors:** Ananda Shankar Bhattacharjee, Amir Mohaghegh Motlagh, Eddie B. Gilcrease, Md Imdadul Islam, Sherwood R. Casjens, Ramesh Goel

**Affiliations:** 1 Department of Civil and Environmental Engineering, University of Utah, Salt Lake City, UT USA; 2 Division of Microbiology and Immunology, Pathology Department, University of Utah School of Medicine, Salt Lake City, UT USA; 3 Department of Biology, University of Utah, Salt Lake City, UT USA; 4 Bigelow Laboratory for Ocean Science, 60 Bigelow Dr., East Boothbay, ME USA; 5 Department of Civil, Environmental, and Construction Engineering, University of Central Florida, 12800 Pegasus Dr., Room 340, Orlando, FL USA

**Keywords:** Bacteriophage, *Delftia tsuruhatensis*, Multidrug resistant, Biofouling, Biofilm, Genome, *Podoviridae*

## Abstract

A lytic bacteriophage RG-2014 infecting a biofilm forming multidrug resistant bacterium *Delftia tsuruhatensis* strain ARB-1 as its host was isolated from a full-scale municipal wastewater treatment plant. Lytic phage RG-2014 was isolated for developing phage based therapeutic approaches against *Delftia tsuruhatensis* strain ARB-1. The strain ARB-1 belongs to the *Comamonadaceae* family of the *Betaproteobacteria* class. RG-2014 was characterized for its type, burst size, latent and eclipse time periods of 150 ± 9 PFU/cell, 10-min, <5-min, respectively. The phage was found to be a dsDNA virus belonging to the *Podoviridae* family. It has an isometric icosahedrally shaped capsid with a diameter of 85 nm. The complete genome of the isolated phage was sequenced and determined to be 73.8 kbp in length with a G + C content of 59.9%. Significant similarities in gene homology and order were observed between *Delftia* phage RG-2014 and the *E. coli* phage N4 indicating that it is a member of the N4-like phage group.

## Introduction

The occurrence and spread of antibiotic resistant bacteria in the environment are regarded as environmental challenges of highest concern in the twenty-first century. ARB bacteria are becoming common, and the Centers for Disease Control and Prevention in the United States estimates more than 23,000 patients die annually due to ARB infections in the US alone [1]. With diminishing opportunities to discover new drugs to combat ARB infections, there is an urgent need to develop alternative therapeutic methods. Phage therapy has been regarded as an alternative to the need of synthesizing new antibiotics [2].

The 10.1601/nm.1802 genus resides in the 10.1601/nm.1773 family of the 10.1601/nm.1616 class and is a Gram negative, short rod-shaped bacterium. 10.1601/nm.1802 species are widely distributed in the environment and have significant biodegradation capability [3, 4]. A recently described species, closely related to 10.1601/nm.1803, 10.1601/nm.1804, has been reported to cause biofouling of bioreactor membranes [5], reverse osmosis membrane filters [6] and heating systems [7]. In addition, 10.1601/nm.1804 has been reported to be the causative agent of catheter-related nosocomial human infections [8, 9]. Previously, we isolated a multi-drug resistant 10.1601/nm.1804 strain 10.1601/strainfinder?urlappend=%3Fid%3DARB+1 from a municipal wastewater treatment plant along with the lytic bacteriophage. We demonstrated phage based therapy to combat biofouling caused by 10.1601/nm.1804 strain 10.1601/strainfinder?urlappend=%3Fid%3DARB+1 with the newly isolated lytic phage as the therapeutic agent [10].

Here, we report the complete genome sequence of the lytic phage specific to 10.1601/nm.1804 10.1601/strainfinder?urlappend=%3Fid%3DARB+1 that we named RG-2014 (it does not infect 10.1601/nm.1802 Cs1–4 or 10.1601/nm.1803 10.1601/strainfinder?urlappend=%3Fid%3DSPH+1 (our unpublished results) [10]. The RG-2014 sequence is annotated and analyzed in order to explore its potential application as an anti-biofilm bio-agent. The host of RG-2014 is multi-drug resistant, using it as a control agent can be an especially appropriate application. The present study is not part of a larger genomic survey.

## Organism information

### Classification and features

The lytic bacteriophage RG-2014 belongs to the Podoviridae family in the order Caudovirales. It is a double-stranded DNA virus that forms 1-2 mm diameter clear plaques when infecting the multidrug resistant bacterium 10.1601/nm.1804 strain 10.1601/strainfinder?urlappend=%3Fid%3DARB+1.

A sample of sludge was obtained from a local wastewater treatment plant, the Central Valley Water Reclamation Facility in Salt Lake City UT, USA. A lytic phage infecting 10.1601/nm.1804 10.1601/strainfinder?urlappend=%3Fid%3DARB+1 was isolated from this sample following a previously described protocol [11, 12]. To remove bacteria and debris the sample was sequentially filtered through 0.45 and 0.2 μm filter membranes [10]. The resulting phage-containing liquid was spotted (without further concentration) on an R2A agar (0.5 g/L protease peptone, 0.5 g/L yeast extract, 0.3 g/L K_2_HPO_4_, 0.05 g/L MgSO_4_·7H_2_O, pH 7) plate containing a lawn of 10.1601/nm.1804 10.1601/strainfinder?urlappend=%3Fid%3DARB+1 [10]. Following incubation of the plates at 37 °C overnight, a clear plaque was picked, followed by the isolation of a second well-separated single plaque on a fresh 10.1601/nm.1804 10.1601/strainfinder?urlappend=%3Fid%3DARB+1 lawn.

**Fig. 1:**
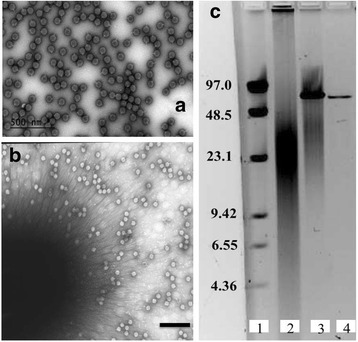
Negative strain transmission electron micrographs of (**a**) RG-2014 virions (scale bar represents 100 nm), (**b**) RG-2014 infecting *D. tsuruhatensis* ARB1 (scale bar represents 1 μm) and (**c**) Pulsed field electrophoresis gel strained with acridine orange; Lane 1, Molecular weight marker (numbers shown are in kbp); Lane 2, 2 μg of DNA from phage RG-2014 virions; lane 3, same as lane 2 with 0.5 μg of phage DNA

As shown in Fig. 1(a) the head of phage RG-2014 virion has a diameter of 85 nm and displays a hexagonal outline implying that it likely possesses icosahedral symmetry. It can also be seen from this transmission electron micrograph, that the virion has a very short tail, indicating that it is a member of the Podoviridae class of viruses. Figure 1(b) shows a micrograph with RG-2014 phage particles attached to a 10.1601/nm.1804 bacterial cell pili; it is not known if such pili may serve as receptor for this phage. Table 1 gives the classification and general features of RG-2014 phage. The genome of the phage is linear double-stranded DNA (dsDNA) that is about 70 kb in length as measured by its mobility during pulsed-field gel electrophoresis (Fig. 1(c)).

**Table 1:** Classification and general features of *Delftia tsuruhatensis* ARB-1 bacteriophage RG-2014

MIGS ID	Property	Term	Evidence code^a^
	Classification	Domain Viruses	TAS [40]
		Kingdom Viruses	TAS [40]
		Phylum: unassigned	TAS [40]
		Class: dsDNA viruses, no RNA phase	TAS [40]
		Order: *Caudovirales*	TAS [40]
		Family: *Podoviridae*	TAS [40]
		Genus: N4likevirus	TAS [40]
		Species: unassigned	
		(Type) strain: RG-2014 (KM879221.2)	
	Gram stain	Not applicable	TAS [40]
	Virion shape	Icosahedral	IDA
	Motility	non-motile	IDA
	Sporulation	Not reported	IDA
	Temperature range	20–38 °C	IDA
	Optimum Temperature	37 °C	IDA
	pH range; Optimum	6.5–7.6	IDA
	Carbon Source	Host cell	IDA
MIGS-6	Habitat	Wastewater	IDA
MIGS-6.3	Salinity	Not reported	
MIGS-22	Oxygen	Facultative aerobic	IDA
MIGS-15	Biotic relationship	Obligate intracellular parasite of *D. tsuruhantensis* ARB-1	IDA
MIGS-14	Pathogenicity	Infective phage of *D. tsuruhantensis* ARB-1	IDA
MIGS-4	Geographic location	Central Valley Water Reclamation Facility, UT, USA	IDA
MIGS-5	Sample collection time	02/01/2011, 11:00 AM	IDA
MIGS-4.1	Latitude	40.7056	IDA
MIGS-4.2	Longitude	111.913953	IDA
MIGS-4.3	Depth	Surface	IDA
MIGS-4.4	Altitude	0 m	

^a^Evidence codes - *IDA* Inferred from Direct Assay, *TAS* Traceable Author Statement (i.e., a direct report exists in the literature)

A one step growth curve was performed with the phage RG-2014 following previously described protocols [10]. The burst size, latent and eclipse period were found to be 150 ± 9 PFU/cell, 10-min, and <5-min, respectively, at 37 °C [10].

**Fig. 2:**
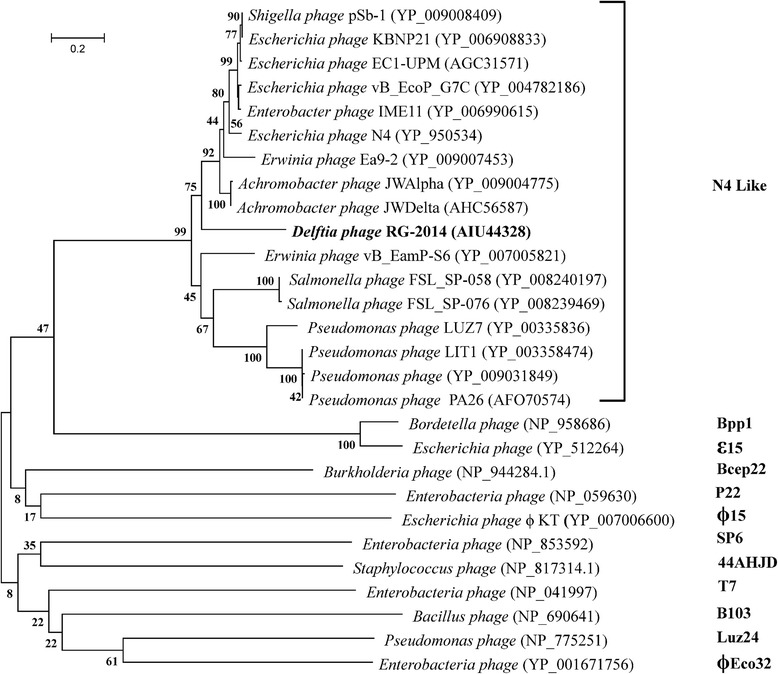
Phylogenetic tree highlighting the position of major coat protein of phage RG-2014 relative to major coat proteins of other hosts. The corresponding GenBank accession numbers for each phage coat protein is indicated in parenthesis. Eleven other types of Podoviridae are included below the N4-like group for comparison. The tree construction used MUSCLE model to align the protein sequences by MEGA (v.5), and the Maximum-likelihood algorithm to construct a distance matrix based on alignment model positions using bootstrap method with 1000 bootstrap replications

The complete genome sequence of the phage RG-2014 was determined. The analysis of the genome clearly shows that it is a member of the N4-like phage group (see below). Grose and Casjens [11] showed that the major capsid proteins (MCPs) of virulent tailed phages parallel the evolution of the nucleotide sequence of the whole phage genome. Phylogeny of the MCPs of selected N4-like phages and other tailed phages shows that the phage RG-2014’s major capsid protein (identified by its similarity that of 10.1601/nm.3093 phage N4, accession no. EF056009) falls robustly within the N4-like phage group (Fig. 2).

## Genome sequencing information

### Genome project history

Phage RG-2014 was isolated in February of 2011, with 10.1601/nm.1804 strain 10.1601/strainfinder?urlappend=%3Fid%3DARB+1 as its host, The genome sequencing and analysis of phage RG-2014 was completed in December of 2016. It is the first genome sequence reported for a lytic phage infecting 10.1601/nm.1804. The purified phage DNA was sequenced with a MiSeq Bench-top DNA sequencer (Illumina, CA) in the High-throughput Genomic Core Facility at the University of Utah. A summary of the phage RG-2014 genome sequencing information is presented below and in the Table 2.

**Table 2:** Project information of *Delftia tsuruhatensis* ARB-1 bacteriophage RG-2014

MIGS ID	Property	Term
MIGS-31	Finishing quality	Closed
MIGS-28	Libraries used	N/A
MIGS-29	Sequencing platforms	Illumina MiSeq Benchtop
MIGS-31.1	Fold coverage	20×
MIGS-30	Assemblers	CLC genomics workbench v. 7.0.3
MIGS-32	Gene calling method	GeneMarkS
	Locus Tag	RG2014
	Genome database release	Genbank
	Genbank ID	KM879221.2
	Genbank Date of Release	Oct, 8, 2014; Mar, 17, 2017 (Corrected genome release date)
	GOLD ID	Go0332698
	BIOPROJECT	PRJNA287956
MIGS 13	Source Material Identifier	Personal culture collection
	Project relevance	Virulence, Bacteriophage based biocontrol

### Growth conditions and genomic DNA preparation

Phage RG-2014 virions were purified from infected 10.1601/nm.1804 10.1601/strainfinder?urlappend=%3Fid%3DARB+1 lysates. Briefly, 0.5 L of cells were grown to 1 × 10^8^ cells per mL in R2A medium at 37 °C with shaking at 150 RPM [10]. The culture was then infected with five RG-2014 phages per cell, followed by incubation for 12 h. After clear cell lysis was observed leading to a cleared culture (the cells lysed), cell debris was removed by centrifugation for 30 mins at 5500×g. Phage virions were then pelleted by centrifugation overnight (>12 h) at 8890×g at 4 °C, and the pellet was re-suspended in SM buffer with Gelatin (5.8 g/L NaCl, 2.0 g/L, MgSO_4_.7H_2_O, 50 mL/L of 1 M Tris-HCl pH 7.5 and 5.0 mL/L of a 5% solution of gelatin). Purified phage virions were obtained by CsCl step gradient centrifugation as described by Earnshaw et al. [12]. The purified phages were stored in SM buffer with gelatin until further use.

The purified RG-2014 virion preparation was used for phage DNA extraction according to the protocol described by Casjens and Gilcrease [13]. Briefly, 400 μL of the CsCl purified phage particles was mixed with 75 μL of lysis buffer (5 μL of 20% SDS, 50 μL 1 M Tris. Cl, 20 μL 0.5 M EDTA, pH = 8) and incubated at 65 °C for 15 min. 50 μL of 5 M potassium acetate was added to the sample and incubated on ice for 1 h. The sample was then centrifuged at 8000×g for 15 min at 4 °C, and the supernatant was carefully transferred into a new 1.5 mL micro-centrifuge tube. After adding 0.9 mL of absolute ethanol to the supernatant and inverting several times, the DNA precipitate was collected by winding it onto the tip of a sterile Pasteur pipette. The DNA precipitate was transferred into a new micro-centrifuge tube, washed with 70% ethanol by inverting a few times, and subsequently pelleted by centrifugation in a microfuge. The DNA pellet was allowed to dry at room temperature for 10–20 min and resuspended in 100 μL of TE buffer (10 mM Tris-Cl pH 7.5 and 1 mM EDTA pH 8.0). About 0.1 μg of the phage DNA was mixed with 5 μL of loading dye and separated by 1% agarose pulsed-field gel electrophoresis (PFGE), with a 1–25-s pulse ramp, a voltage of 6.0 V/cm with an angle of 120° for 24 h at a constant temperature of 14 °C on a CHEF DR III system (Bio-Rad, USA). After completion of electrophoresis the gel was stained with ethidium bromide (Molecular Probes, USA) and visualized under CHEM DOC gel documentation system (Bio-Rad, USA).

### Genome sequencing and assembly

Approximately 8 million paired-end reads with an average length of 300 bp were generated using a MiSeq Bench-top DNA sequencer (Illumina, CA). The reads were interleaved and trimmed based on a Phred score of 28 and a minimum post-trimming average length of 290 bp on the CLC Genomics Workbench 7.0.4 (CLC Bio, Denmark). The trimmed reads were de novo assembled on the CLC Genomics Workbench 7.0.4 with the following criteria: word size, 20 bp; automatic bubble size, 50 bp; minimum contig length, 200 bp as described in Bhattacharjee et al. [10].

The termini of the virion chromosome were determined by dideoxynucleotide Sanger sequencing [14] using the virion DNA as a template using the following primers which direct sequencing runs off the two ends as follows; right end, 5′-TGCTTCATGATCTTCAGTCC-3′ and left end, 5′-GAAGGCATCAGCATGTTCAG-3′.

### Genome annotation

Glimmer [15] was used to identify the open reading frames and GeneMarkS [16] for predicting genes. The predicted genes were used to search the NCBI non-redundant database, the conserved domain database, the Cluster of Orthologous Groups database and the InterPro database and were annotated using Blast2GO 2.5.0 [17]. Automated annotation performed by Blast2GO 2.5.0 was manually curated by individually analyzing each predicted gene using BLAST against NCBI nr database with minimum e-value cut off of 10^−3^ [18]. ARAGORN [19] and tRNAScanSE [20] were used for detection of transfer RNA genes. The complete annotated genome sequence is available in Genbank under the accession number KM879221.

## Genome properties

The lytic phage RG-2014’s complete genome size was found to be 73,882 bps that includes 450 bp direct terminal repeats (we note that, when it has been examined, the genomes of other N4-like phages invariably have several hundred bp terminal repeats)with a G + C content of 59.9%. The annotation includes 88 putative protein coding ORFs and no tRNAs (Table 3). Predicted proteins were classified in COG functional categories [21, 22] using the WebMGA web server for metagenome analysis [23]. The number of predicted genes and the relative percentage of phage genes associated with the 25 general functional COG categories are described in Table 4. Twenty-eight (31.8%) of the 88 genes in the RG-2014 phage genome were assigned a putative function based on significant sequence similarity to genes of known functionality in the NCBI database. Twenty-one (23.8%) genes encode putative proteins that were assigned to the conserved hypothetical protein category. Additionally, 40 predicted genes (44.3%) had no similarity to genes in the current database, and their products were classified as hypothetical proteins (Table 5). Annotation using the CDD on the NCBI server was also performed and is presented in Table 6.

**Table 3:** Genome statistics

Attribute	Value	% of Total^a^
Genome size (bp)	73,882	100.00
DNA Coding (bp)	69,793	93.90
DNA G + C (bp)	44,247	59.90
DNA scaffold	0	0.00
Total genes	88	100.00
Protein-coding genes	88	100.00
RNA genes	0	0.00
Pseudo genes	0	0.00
Genes in internal clusters	0	0.00
Genes with function prediction	21	23.86
Genes assigned to COGs	10	9.09
Genes with Pfam domains	12	13.64
Genes with signal peptides	2	2.27
Genes with transmembrane helices	13	14.77
CRISPR repeats	0	0.00

^a^The total is based on either the size of the genome in base pairs or the total number of protein coding genes in the annotated genome

**Table 4:** Number of genes associated with the 25 general COG functional categories

Code	Value	% age^a^	Description
J	0	0	Translation
A	0	0	RNA processing and modification
K	2	2.27	Transcription
L	2	2.27	Replication, recombination and repair
B	0	0	Chromatin structure and dynamics
D	0	0	Cell cycle control, mitosis and meiosis
Y	0	0	Nuclear structure
V	0	0	Defense mechanisms
T	0	0	Signal transduction mechanisms
M	1	1.14	Cell wall/membrane biogenesis
N	1	1.14	Cell motility
Z	0	0	Cytoskeleton
W	0	0	Extracellular structures
U	0	0	Intracellular trafficking and secretion
O	0	0	Posttranslational modification, protein turnover, chaperones
C	0	0	Energy production and conversion
G	0	0	Carbohydrate transport and metabolism
E	0	0	Amino acid transport and metabolism
F	2	2.27	Nucleotide transport and metabolism
H	0	0	Coenzyme transport and metabolism
I	0	0	Lipid transport and metabolism
P	0	0	Inorganic ion transport and metabolism
Q	0	0	Secondary metabolites biosynthesis, transport and catabolism
R	2	2.27	General function prediction only
S	1	1.14	Function unknown
–	77	87.5	Not in COGs

^a^The total is based on the total number of protein coding genes in the annotated genome

**Table 5:** *Delftia* phage RG-2014 gene prediction

Gene	Strand	Number of codons	Predicted function	Organism with best match	N4 gene^a^	Gene accession no.	% Id^b^	E-value^b^
1	+	101	Conserved hypothetical protein	*Erwinia* phage Ea9–2	–	AIU44254	32	0.002
2	+	139	Conserved hypothetical protein	*Achromobacter* phage JWdelta	2	AHC56518	36	2e-21
3	+	121	Hypothetical protein	–	–	–	–	–
4	+	122	Conserved hypothetical protein	*Roseovarius* sp. phage 1	14	CBW47037	57	3e-45
5	+	109	Hypothetical protein	–	–	–	–	–
6	+	115	Hypothetical protein	–	–	–	–	–
7	–	104	Hypothetical protein	–	–	–	–	–
8	+	105	Hypothetical protein	–	–	–	–	–
9	+	50	Hypothetical protein	–	–	–	–	–
10	+	69	Hypothetical protein	–	–	–	–	–
11	+	186	Conserved Hypothetical protein	*Pithovirus sibericum*	–	YP 009001006	32	3e-22
12	+	285	Conserved hypothetical protein	*Achromobacter* sp.	–	CYTR01000018	38	2e-26
13	+	108	Conserved hypothetical protein	*Escherichia* phage phAPEC8	3	YP_007348409	29	3e-04
14	+	137	Hypothetical protein	–	–	–	–	–
15	+	89	Hypothetical protein	–	–	–	–	–
16	+	44	Hypothetical protein	–	–	–	–	–
17	+	77	Hypothetical protein	–	–	–	–	–
18	+	142	Conserved hypothetical protein	*Achromobacter* phage øAxp-3	–	YP_009148381	55	3e-37
19	+	193	Hypothetical protein	–	–	–	–	–
20	+	76	Conserved hypothetical protein	*Pseudomonas* phage PPpw-3^**c**^	–	YP_008873216	40	5e-09
21	+	217	Hypothetical protein	–	–	–	–	–
22	+	272	RNA polymerase I subunit	*Erwinia* vB EamP Rexella	15	ANJ65251	54	1e-102
23	+	432	RNA polymerase II subunit	*Erwinia* phage Ea9–2	16	AAL71577	47	1e-135
24	+	181	Virion decoration protein	*Achromobacter* phage øAxp-3	17	YP_009208670	36	1e-10
25	+	157	Hypothetical protein	–	–	–	–	–
26	+	155	Hypothetical protein	–	–	–	–	–
27	+	122	Hypothetical protein	–	–	–	–	–
28	+	82	Hypothetical protein	–	–	–	–	–
29	+	80	Hypothetical protein	–	–	–	–	–
30	+	115	Hypothetical protein	–	–	–	–	–
31	+	242	Hypothetical protein	–	–	–	–	–
32	+	209	Hypothetical protein	–	–	–	–	–
33	+	359	Conserved hypothetical protein	*Erwinia* phage Ea9–2	24	AHI60096	46	7e-104
34	+	127	Conserved hypothetical protein	*Achromobacter* phage øAxp-3	–	YP009208682	41	7e-11
35	+	92	Hypothetical protein	–	–	–	–	–
36	+	405	Conserved hypothetical protein	*Escherichia* phage N4	25	ABK54394	39	1e-86
37	+	170	dCTP deaminase	*Escherichia* phage Bp4	26	AHN83412	51	4e-53
38	+	78	Hypothetical protein	–	–	–	–	–
39	+	124	Hypothetical protein	–	–	–	–	–
40	+	140	Hypothetical protein	–	–	–	–	–
41	+	169	Hypothetical protein	–	–	–	–	–
42	+	121	Hypothetical protein	–	–	–	–	–
43	+	103	Hypothetical protein	–	–	–	–	–
44	+	73	Hypothetical protein	–	–	–	–	–
45	+	317	Thymidylate synthase	*Salmonella* phage SEGD1^**c**^	–	KU726251	48	2e-101
46	+	104	Conserved hypothetical protein	*Escherichia* phage N4	35	YP_950513	59	1e-36
47	+	135	Conserved hypothetical protein	*Paenibacillus* phage PG1^**c**^	–	YP_008129928	66	5E-54
48	+	197	Nucleotide pyrophospho-hydrolase	*Pseudomonas* phage PaMx74^**c**^	–	YP_009199508	33	3e-13
49	+	436	DNA helicase	*Achromobacter* phage JWdelta	37	AHC56567	48	4e-137
50	+	172	Conserved hypothetical protein	*Achromobacter* phage JWalpha	38	YP_009004756	34	2e-27
51	+	884	DNA polymerase	*Escherichia* phage N4	39	ABK54408	60	0.0
52	+	127	Hypothetical protein	–	–	–	–	–
53	+	286	Conserved Hypothetical protein	*Nitrincola* phage 1 M3–16	–	YP 009037286	47	1e-12
54	+	327	Conserved hypothetical protein	*Escherichia* phage G7C	41	AEL79653	45	7e-97
55	+	724	DNA primase	*Achromobacter* phage øAxp-3	42	ALA45517	62	0.0
56	+	249	Conserved hypothetical protein	*Escherichia* phage N4	43	ABK54413	57	3e-100
57	+	253	Single-stranded DNA-binding protein	*Erwinia* phage S6	44	AEJ81593	38	5e-37
58	+	372	Conserved hypothetical protein	*Salmonella* phage FSL_SP-076	–	YP_008240188	43^**4**^	4e-24
59	+	61	Hypothetical protein	–	–	–	–	–
60	+	65	Hypothetical protein	–	–	–	–	–
61	+	235	Hypothetical protein	–	–	–	–	–
62	+	102	Hypothetical protein	–	–	–	–	–
63	+	59	Hypothetical protein	–	–	–	–	–
64	+	98	Conserved Hypothetical protein	*Bacillus* phage SP-10	–	YP 007003301	40	3e-10
65	+	288	Possible transcriptional regulator	*Burkholderia* phage AH2^**c**^		AEY69538	38	5e-44
66	+	110	Hypothetical protein	–	–	–	–	–
67	+	172	Hypothetical protein	*Deftia* phage øW-14^**3**^	–	YP_003359016	39^**e**^	1e-10
68	–	3413	Virion RNA polymerase	*Achromobacter* phage øAxp-3	50	ALA45523	42	0.0
69	–	712	Lysozyme-like domain virion structural protein	*Escherichia* phage ECBP1	51	AFR52010	25	5e-18
70	–	135	Conserved hypothetical protein	*Achromobacter* phage JWdelta	–	AHC56583	75	2e-38
71	–	921	Conserved hypothetical protein	*Achromobacter* phage øAxp-3	53	ALA45526	36	1e-168
72	–	300	Virion structural protein	*Escherichia* phage N4	54	AAO24827	50	2e-101
73	–	265	Conserved hypothetical protein	*Achromobacter* phage øAxp-3	55	ALA45528	38	3e-47
74	–	411	Major capsid protein	*Achromobacter* phage øAxp-3	56	ALA45529	66	0.0
75	–	281	Conserved hypothetical protein	*Escherichia* phage IME11	57	AFV29058	38^**d**^	7e-42
76	–	116	Hypothetical protein	*Erwinia* phage S6	58	YP_007005822	71^**e**^	0.006
77	–	138	Conserved Hypothetical protein	*Roseovarius* sp. 217 phage 1	–	CBW47064	28	0.002
78	–	766	Portal protein	*Erwinia* phage Frozen	59	ANJ65209	59	0.0
79	–	170	Lysis / possible Rz-like spanin	*Achromobacter* phage JWalpha	60	AHC94031	40	4e-21
80	–	201	Lysis / N-acetylmuramidase	*Escherichia* phage G7C	61	AEL79672	52	7e-71
81	–	108	Conserved hypothetical protein	*Escherichia* phage N4	63	ABK54424	34	1e-17
82	–	416	Conserved hypothetical protein	*Achromobacter* phage øAxp-3	64	ALA45537	64	0.0
83	–	1388	Tail sheath and receptor binding virion protein	*Achromobacter* phage øAxp-3	65	ALA45538	51	0.0
84	–	140	Hypothetical protein	–	–	–	–	–
85	–	234	Possible virion appendage protein	*Erwinia* phage Ea9–2	66	AHI60147	44^**d**^	2e-67
86	–	536	Large terminase subunit	*Escherichia* phage ECBP1	68	AFR52033	61	0.0
87	–	228	Conserved hypothetical protein	*Escherichia* phage N4	69	ABK54430	46	2e-61
88	–	340	Conserved hypothetical protein	*Achromobacter* phage øAxp-3	49	ALA45543	34	1e-17

^a^ *E. coli* phage N4 is the best characterized and therefore the prototypical member of this phage group

^b^% identity and e-value determined by BLASTp at NCBI web site; unless otherwise noted, values are listed if the patch of similarity includes ≥60% of the protein

^c^All phages in this column are in the N4-like group except AH2, øW-14, SEGD1, PG1, PaMx74 and PPpw-3

^d^Sequence similarity only in N-terminal region

^e^Sequence similarity only in C-terminal region

**Table 6:** *Delftia* phage RG 2014 annotation using conserved domain database*

Gene	Evidence	E value	Bit Score	Accession
4	cl10259 superfamily	2.22E-55	167.72	Cl10259
12	MTTB superfamily	0.004977	36.9774	Cl15385
15	MDR superfamily	0.0037317	33.0936	Cl16912
22	Pha00452	1.96E-05	44.2438	Pha00452
23	RNA_pol superfamily	4.77E-09	56.9554	Cl20211
24	Big_2	0.00242	34.2896	Pfam02368
24	Big_2 superfamily	3.49E-07	39.6898	Cl02708
24	Cog5492	3.72E-09	53.2664	Cog5492
33	Aaa	6.72E-05	41.3627	Cd00009
33	ABC_atpase superfamily	6.72E-05	41.3627	Cl21455
36	Vwfa	0.0009673	38.3158	Cd00198
36	Vwfa superfamily	1.20E-20	85.5169	Cl00057
36	DUF2201_N superfamily	9.26E-31	117.611	Cl16157
37	Trimeric_dutpase	3.53E-13	60.5857	Cd07557
37	Trimeric_dutpase superfamily	2.62E-23	89.0534	Cl00493
45	TS_Pyrimidine_hmase	5.70E-91	268.76	Cd00351
45	TS_Pyrimidine_hmase superfamily	5.17E-137	387.525	Cl19097
48	NTP-ppase superfamily	0.002418	35.1816	Cl16941
49	ABC_atpase superfamily	2.77E-17	78.3824	Cl21455
49	Uvrd_C_2	8.48E-08	47.1547	Pfam13538
49	Uvrd_C_2 superfamily	8.48E-08	47.1547	Cl22491
49	Aaa_30	2.77E-17	78.3824	Pfam13604
49	Cog1112	9.34E-05	43.4113	Cog1112
51	DNA_pol_A superfamily	1.80E-26	110.198	Cl02626
51	DNAq_like_exo superfamily	0.0005505	40.4172	Cl10012
55	Prict_1	1.28E-07	47.6526	Pfam08708
55	Prict_1 superfamily	1.28E-07	47.6526	Cl07362
56	ABC_atpase superfamily	0.0005674	38.4072	Cl21455
63	Prk14085	0.0005556	34.1837	Prk14085
64	DUF2829	1.18E-16	66.5176	Pfam11195
64	DUF2829 superfamily	1.18E-16	66.5176	Cl12744
65	Parbc	0.0004658	37.3039	Pfam02195
65	Parbc superfamily	0.0003729	37.2839	Cl02129
66	DUF1178	0.0021343	34.0766	Pfam06676
67	Extradiol_Dioxygenase_3B_like superfamily	0.0057676	34.7714	Cl00599
69	Lt_gewl	1.36E-18	80.5286	Cd00254
69	Lysozyme_like superfamily	1.36E-18	80.5286	Cl00222
70	Polyadenylate-binding_protein_3	0.0067594	34.0122	Tigr01628
72	DUF3584	0.0060894	36.9891	Pfam12128
74	Hypothetical_protein	5.26E-76	237.638	Tigr04387
74	P22_coatprotein superfamily	5.26E-76	237.638	Cl22542
78	Cog4913	0.001198	41.1603	Cog4913
79	Prk09039	0.000734	37.6381	Prk09039
80	Glyco_hydro_108 superfamily	9.31E-23	86.0288	Cl09583
80	PG_binding_3 superfamily	0.0001066	38.2277	Cl09627
86	COG5362 superfamily	3.02E-08	51.3532	Cl02216
88	Phage_gp49_66	2.28E-21	85.3759	Pfam13876
88	Phage_gp49_66 superfamily	2.28E-21	85.3759	Cl10351

*Evidence of gene functions provided by blast analysis using conserved domain database (*e*-value ≤10^−5^)

## Insights from the genome sequence

**Fig. 3:**
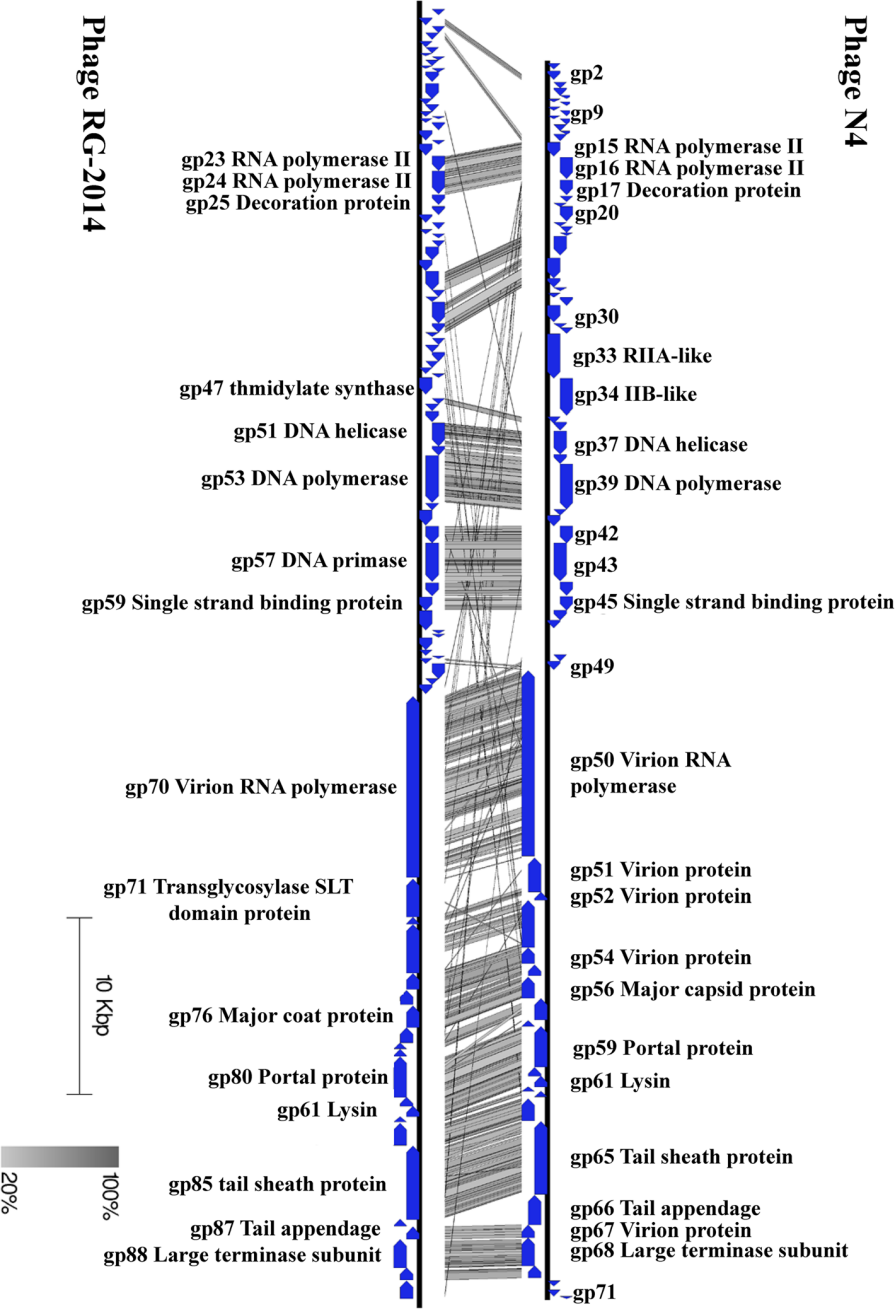
Whole genome comparison of *Delftia* phage RG-2014 (KM872991.2) phage to *E. coli* phage N4 (NC_008720). The Figure was generated with Easyfig [38]. Genomes were aligned using Easyfig [38]. The functions of genes in phage N4 are shown above and predicted functions of RG-2014 genes are indicated below the maps

The phylogenetic tree of MCPs in Fig. 2 indicates that phage RG-2014 is most closely related to the group of phages typified by 10.1601/nm.3093 *phage* N4 (NC_008720) [13, 24–28]. In addition their hosts, 10.1601/nm.3093 K-12 and 10.1601/nm.1804 strain 10.1601/strainfinder?urlappend=%3Fid%3DARB+1 belong to the same phylum 10.1601/nm.808. Table 1 summarizes the classification and general features of the phage RG-2014. BLAST searches using the 10.1601/nm.1802 *phage* RG-2014 genome as a probe was undertaken to confirm this notion. Genome comparisons with 10.1601/nm.3093 *phage* N4 (NC_008720) were performed, and significant similarities in gene homology and order were observed between phages RG-2014 and N4 (Table 5 and Fig. 3). The phage RG-2014 genome shows mosaicism that is typical of tailed phages, with (for example) some regions displaying close relatedness to phage N4 (Fig. 3). Mosaicism in bacteriophage genomes is a well-known phenomenon wherein regions of high similarity are interspersed with less related or unrelated regions. These mosaic patterns in bacteriophage genomes corroborate the theory that horizontal gene transfer plays a significant role in phage evolution [29–31].

10.1601/nm.3093 *phage* N4 does not depend upon its host’s RNA polymerase to transcribe its early and middle genes. But encodes its own set of two RNAPs. These are encoded by three genes, one for the early RNAP and the two subunits of the middle gene transcribing RNAP [28, 32]. The host’s RNAP transcribes the N4 late genes. A striking and unique feature of this type of phage is that a unique single-subunit vRNAP is carried in the virion. vRNAP is encoded by N4 gene *50* and is injected into the host cell with the DNA where it transcribes the phage’s early genes. The RNAPII that transcribes the middle genes and is encoded by the two N4 genes *15* and *16*. The RG-2014 genome harbors three genes that are homologues of the N4 RNAP genes, *68, 22* and *23*, respectively. The closest relatives of these RG-2014 genes are present in N4-like phages 10.1601/nm.1738 phage øAxp-3, *Erwina* phage Frozen, and *Erwina* phage Ea9–2, respectively (Table 5).

Most of the N4 like phages have been shown to harbor between 1 and 3 genes encoding tRNA. Paepe et al. [33] and Bailey-Bechet et al. [34] suggesting, virulent phages harbor more tRNA genes than temperate phages to ensure optimal translation leading to faster replication. However, the phage RG-2014 genome lacks transfer RNA genes, suggesting that the phage is highly adapted to its host 10.1601/nm.1804 10.1601/strainfinder?urlappend=%3Fid%3DARB+1, with regard to codon usage, allowing it to translate its genes efficiently without the need of synthesizing its own tRNAs [24]. To support our finding average codon usage bias was calculated for the phage RG-2014 and 10.1601/nm.1804 CM13 (NZ_CP017420), a close representative of the host 10.1601/nm.1804 10.1601/strainfinder?urlappend=%3Fid%3DARB+1. The average codon usage bias calculation was performed using CodonO web server (http://sysbio.cvm.msstate.edu/CodonO/) [35]. 10.1601/nm.1804 CM13 (NZ_CP017420) and phage RG-2014 had similar average codon usage bias of 0.440141 and 0.406048, respectively, suggested the phage was adapted to its host.

There are two known types of virion assembly gene arrangements in the N4-like phages. First, those like phage N4 that have a single contiguous gene cluster that encodes all of the known structural genes and lysis proteins except the head decoration protein (N4 gene *17*). Second, typified by 10.1601/nm.2552 phage LIT1 in which several tail genes are present inside the replication gene cluster [25, 36]. Phage RG-2014 carries a set of homologous genes, including the separate decoration protein gene (RG-2014 gene *24*), that have the phage N4 type organization. By homology to those of N4 [36], RG-2014 genes *24, 68, 69, 71–78, 83* and *85* encode virion structural proteins.

Phage RG-2014 makes clear plaques and carries no genes that encode proteins (such as integrase or protelomerase) that might suggest a temperate lifestyle. In addition, we also recently showed that the database of bacterial genome sequences has grown to a point where relatives of essentially all known temperate phages can be found as prophages present in the reported genome sequences of their hosts [37]. Thus, absence of *closely related* homologous genes (the MCP gene was used in that study) in closely related host genomes of the same bacterial family is strong evidence that a phage is virulent; related prophages would be found to encode such a gene if the phage in question were temperate. In fact no genes that are closely related to MCP of the phage RG-2014 are present in the current bacterial sequence database. The closest MCP gene relatives in prophages are from the distantly related bacterial genera 10.1601/nm.1414, 10.1601/nm.3232 and 10.1601/nm.2765 whose encoded homologous proteins are only 47–56% identical to the amino acid sequence of phage RG-2014 MCP. The latter gene matches are found (when the sequence contigs are sufficiently large for such a determination) to be present in rather distantly related prophages that have other similarities to the N4-like phages including a prophage encoded vRNAP, suggesting that there are currently undescribed temperate phages that are very distantly related to the N4-like phage group (our unpublished observation). Nonetheless, among the 143 currently available genomes from the 10.1601/nm.1773 bacterial family (including eight 10.1601/nm.1802 genomes) the best-encoded protein matches have only 22% identity to the phage RG-2014 MCP. We conclude that phage RG-2014 is virulent.

**Fig. 4:**
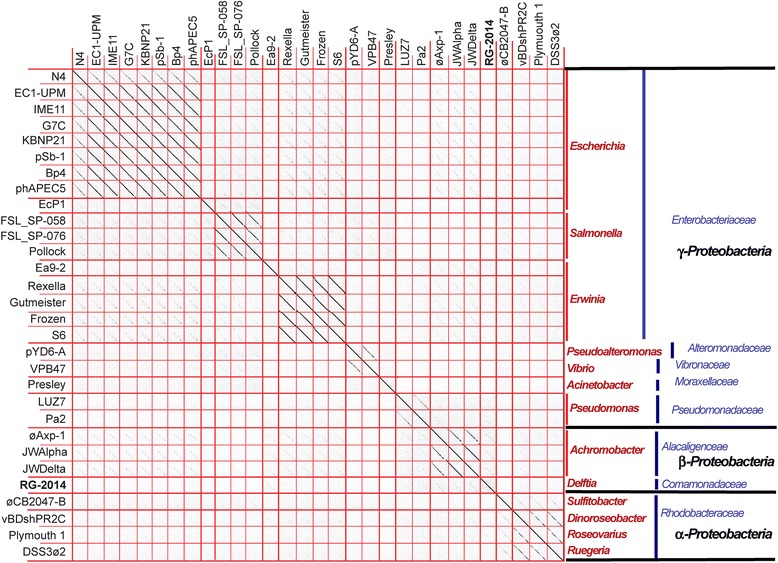
Dotplot of N4-like phage genomes. Phage genomes were arranged in the same orientation and a dot plot was constructed by Gephard [39] with a word length setting of 11. The phages in the figure include the current extant diversity among the N4-like phages; those that are not included are very similar to one of the phages that is included (their sequences are all in GenBank and can be retrieved by searching with their names). In the plot thin red lines separate the phage genomes, and thick red lines separate the most clearly delineated subtypes. At the right, the genus (red text), family (black text) and class (blue text) of each phage’s host bacteria are indicated; vertical very thick red lines on the right indicate phages that infect the same host genus, and very thick blue lines mark host families

The N4-like phage group is clearly well separated from the other known tailed bacteriophages [11, 28], but the taxonomic status of different phages within the group remains less understood. Unlike some other tailed phage types, the N4-like phages include members that infect a wide range of bacterial hosts in the *Alphaproteobacteria*, *Betaproteobacteria* and *Gammaproteobacteria* classes [25, 28]. Fig. 4 shows a dotplot of a diverse sample of N4-like phage genomes that illuminates several aspects of the phages in this group (no diagonal lines are present when comparison is with other tailed phage types, data not shown). First, phage RG-2014 is not particularly closely related to any of the other currently known N4-like phages; its closest, but nonetheless rather distant, relatives are 10.1601/nm.1738 phages JWDelta, JWAlpha and øAxp-1. We note that these four phages infect members of the *Βetap* 10.1601/nm.808. A second conclusion that can be drawn from fig. 4 is that genome similarity within this group of phages generally parallels the relatedness of their hosts. The various subtypes of the N4-like phage group (separated by thick red lines in the figure) are usually restricted to single genus; the one current exception to this rule is the relatively close relationship between 10.1601/nm.2945 phage 10.1601/strainfinder?urlappend=%3Fid%3DVPB+47 and 10.1601/nm.8928 phage pYD6-A. It thus appears that recent “jumping” of these phages between taxonomically distant hosts is not common. On the other hand, more than one N4-like phage subtype can infect a given host genus; for example, 10.1601/nm.3092 and 10.1601/nm.3165 N4-like phages are clearly present as two subtypes (e.g. the 10.1601/nm.3092 N4/EcP1 and 10.1601/nm.3165 Ea9–2/S6 pairs). More distant host relationships are complex. Very weak diagonal similarity lines are present when the 10.1601/nm.3092 (phage N4 subtype), 10.1601/nm.3165 and *Achromobacter* N4-like phages are compared. These could tentatively correspond to members of the proposed *Enquatravirinae* subfamily [28].

## Conclusions

The 10.1601/nm.1804 infecting phage RG-2014 belongs to the *Podoviridae* viral family. The phage RG-2014 genome sequence shows significant synteny and sequence similarity to 10.1601/nm.3093 bacteriophage N4 and other members of the N4-like group of tailed phages; this clearly demonstrates phage RG-2014’s membership in this group. Our analysis confirms that phages in the virulent N4-like group are widely present in the wild. The members of the N4-like group infect bacterial hosts in several classes within the 10.1601/nm.808 phylum. Their virulent nature, widespread distribution and efficient infection suggest that members of this group will be useful in many bacterial control situations.

## Abbreviations

ARB: Antibiotic Resistant Bacteria
COG: Cluster of Orthologous Groups
ORF: Open reading frame
RNAP: RNA polymerase
